# Macrophage Plasticity and Regulatory Networks During the Transition from Inflammation to Fibrosis in the Kidney

**DOI:** 10.3390/life16010052

**Published:** 2025-12-29

**Authors:** Yehun Moon, Jintaek Hong, Jinwoo Chung, Jea-Hyun Baek

**Affiliations:** Laboratory of Inflammation Research, School of Life Science, Handong Global University, Pohang 37554, Republic of Korea; 22000248@handong.ac.kr (Y.M.); 22000812@handong.ac.kr (J.H.); 21900683@handong.ac.kr (J.C.)

**Keywords:** macrophage plasticity, renal fibrosis, metabolic reprogramming

## Abstract

Kidney fibrosis represents the final common pathway of nearly all progressive renal diseases, linking acute kidney injury (AKI) and chronic kidney disease (CKD) through a maladaptive repair process. Regardless of etiology, persistent inflammation and excessive extracellular matrix (ECM) deposition drive irreversible structural distortion and functional decline in the kidney. Among cellular mediators, macrophages occupy a central role across the continuum from acute injury to fibrosis, orchestrating both tissue injury and repair through dynamic transitions between pro-inflammatory (M1) and pro-fibrotic (M2) states in response to local cues. Here, we synthesize macrophage-driven mechanisms of renal fibrosis, emphasizing recruitment, infiltration, and local proliferation mediated by chemokine–receptor networks and mechanosensitive ion channels. In addition, in this review paper, we provide an overview on the dual roles of macrophages in acute inflammation and chronic remodeling through key cytokine signaling pathways (TLR4/NF-κB, IL-4/STAT6, TGF-β/Smad, IL-10/STAT3), highlighting how metabolic reprogramming, mechanochemical feedback via Yes-associated protein (YAP)/transcriptional coactivator with PDZ-binding motif (TAZ) signaling, and epigenetic modulators collectively stabilize the fibrotic macrophage phenotype. Also, emerging insights into mitochondrial dysfunction, succinate–succinate receptor 1 (SUCNR1) signaling, and autophagy dysregulation reveal the metabolic basis of macrophage persistence in fibrotic kidneys. Understanding these multilayered regulatory circuits offers a framework for therapeutic strategies that selectively target macrophage-dependent fibrogenesis to halt the transition from acute injury to chronic renal failure.

## 1. Introduction

Kidney diseases represent a growing global health challenge, affecting hundreds of millions of individuals across diverse populations. These conditions encompass a spectrum ranging from acute kidney injury (AKI), featuring an abrupt decline in renal function [1,2], to chronic kidney disease (CKD) through a gradual loss of kidney structure and performance over prolonged periods [3,4,5].

Renal fibrosis is the most consistent pathological outcome that drives the progression from early renal injury to end-stage renal disease (ESRD), regardless of the diverse initial etiologies, which include diabetes, hypertension, trauma, infection, and iatrogenicity. Rather than serving a successful defensive function, fibrosis represents an active, yet maladaptive, repair response characterized by the excessive accumulation of extracellular matrix (ECM) components and the proliferation of myofibroblasts beyond normal regulatory control [6,7,8]. This abnormal remodeling manifests as glomerulosclerosis and tubulointerstitial fibrosis, structural changes that profoundly impair the kidney’s filtration capacity and disrupt its crucial role in fluid and electrolyte balance [4,9]. The increasing severity of fibrosis correlates strongly with the progression to end-stage renal failure, making it one of the most reliable indicators of irreversible kidney damage [9,10]. Understanding this fibrotic progression remains a significant challenge in nephrology and requires a profound dissection of the molecular and cellular mechanisms that govern this pathological repair process. Recent publications utilizing single-cell transcriptome analysis of injured kidneys have been particularly revealing, uncovering a previously unrecognized diversity among macrophages involved in kidney fibrosis (Table 1).

A wide range of immune and stromal cell populations enter or emerge within the injured kidney, fundamentally influencing the transition from inflammation to fibrosis [11,12,13]. Especially, monocytes and macrophages are positioned at the center of this fibrotic environment. Previous studies have demonstrated that macrophages are key regulators, mediating the complex interplay between inflammatory responses and tissue remodeling. Macrophages that continuously infiltrate and reside within chronically damaged kidney tissue secrete various cytokines and growth factors. They promote renal parenchymal cell injury, drive the activation of myofibroblasts, and actively shape the fibrotic microenvironment [14,15,16].

The ability of macrophages to orchestrate these diverse functions stems from their remarkable phenotypic plasticity. Macrophages dynamically alter their functional state in response to signals from the surrounding microenvironment [15,17]. The classical M1 (pro-inflammatory) and the alternative M2 (anti-inflammatory/tissue repair) polarization model provided an initial framework for understanding versatile macrophage functions. During the early stages of injury, M1-like macrophages amplify inflammatory responses and contribute to tissue damage [18,19,20]. As injury persists, these cells adopt M2 phenotypes that support wound resolution or promote excessive ECM deposition, with the latter contributing to chronic fibrotic scarring [21,22]. However, within the complex and persistent pathological setting of renal fibrosis, macrophages exist across a diverse functional spectrum rather than two distinct populations. They often exhibit mixed phenotypes with overlapping inflammatory and pro-fibrotic characteristics. Their complex states are determined by multilayered regulation involving cytokine signaling, metabolic reprogramming, and epigenetic modulation [23,24,25].

Historically, the functional shift of macrophages has been characterized by surface marker expression, such as the classical M1/M2 polarization paradigm. However, emerging studies highlight that macrophage plasticity is not merely a transcriptional phenomenon but is deeply rooted in profound metabolic remodeling at the cellular level [26,27]. Mechanistically, metabolic reprogramming represents the ability of macrophages to deliberately rewire their energy-producing pathways, including glycolysis, the tricarboxylic acid (TCA) cycle, and fatty acid oxidation (FAO), to meet specific functional demands [24]. Beyond being a consequence of activation, metabolic reprogramming actively shapes macrophage fate and function. For example, arginine metabolism in the urea cycle is also converted to ornithine and polyamines. This conversion promotes cell proliferation and tissue repair [28,29].

**Table 1:** The table shows a list of recent publications utilizing single-cell transcriptome analysis of injured kidneys. Abbreviations: AKI (acute kidney injury), CaOx (calcium oxalate crystal nephropathy), CKD (chronic kidney disease), CN (crystal nephropathy), DKD (diabetic kidney disease), FACS (fluorescence-activated cell sorting), IMQ (imiquimod-induced), IRI (ischemia–reperfusion injury), LN (lupus nephritis), scRNA-seq (single-cell RNA sequencing), snRNA-seq (single-nucleus RNA sequencing), STZ (Streptozotocin), UUO (unilateral ureteral obstruction).

Studies	Omics Type	Platform	Cell Isolation Procedure	Disease Type	Injury Model (dpi)	Reference
Conway et al., 2020	scRNA-seq (droplet)	10x Genomics 3′ v2	Whole kidney + FACS	CKD	UUO (0, 1, 3, 7)	[30]
	scRNA-seq (plate)	SMART-seq2				
Yao et al., 2022	scRNA-seq	10x Genomics 3′	Whole kidney + FACS	AKI	IRI (1)	[31]
Fan et al., 2024	scRNA-seq	Integrated datasets	Human kidney	CKD	Human CKD (N/A)	[32]
Ji et al., 2024	scRNA-seq	10x Genomics 3′	Whole kidney	DKD (CKD)	STZ (N/A)	[33]
Liu et al., 2024	scRNA-seq	10x Genomics 3′ v3	Whole kidney + FACS	AKI	UUO + IRI (2, 3, 7)	[34]
Jin et al., 2025	scRNA-seq	10x Genomics 3′ v3	Whole kidney	CN (CKD)	CaOx (0, 1, 3, 5, 7)	[35]
Xing et al., 2025	Spatial transcriptomics	Stereo-seq	Whole kidney (single nuclei)	LN	IMQ (N/A)	[36]
	snRNA-seq	MGI DNBelab C Series				
Zheng et al., 2025	scRNA-seq	Integrated datasets	Whole kidney	AKI	IRI (7)	[37]

In this review, we provide an overview of how macrophages shape the transition from AKI to fibrosis by integrating inflammatory, metabolic, and mechanical cues within the renal microenvironment. We outline the processes that regulate macrophage recruitment and activation and summarize how these cells adopt pro-inflammatory or pro-fibrotic phenotypes that influence tissue outcomes. Particular attention is given to regulatory layers involving cytokine signaling, metabolic adaptation, mechanotransduction, and epigenetic or RNA-mediated control, all of which contribute to the stabilization of macrophage states during fibrosis. A clearer understanding of how these pathways interact may reveal therapeutic opportunities to limit macrophage-driven fibrogenesis and slow the progression from AKI to CKD.

## 2. Macrophage Accumulation During AKI-CKD Transition

Macrophage accumulation is a hallmark of renal inflammation and fibrosis, driven by three interconnected mechanisms: recruitment, infiltration, and local proliferation. Circulating monocytes derived from bone marrow serve as the primary source of infiltrating macrophages.

### 2.1. Chemokine-Mediated Recruitment

The recruitment of monocytes into the injured kidney is orchestrated by specific chemokines and their receptors, most notably the CCL2–CCR2 axis, alongside CXCL16–CXCR6, CCL5–CCR5, and fractalkine (CX3CL1)–CX3CR1 [38,39]. Recent single-cell analyses show that the CXCL1–CXCR2 and CCL2/CCL3–CCL2 axis rapidly recruits S100A8/A9^hi^ Ly6C^hi^ inflammatory monocytes as the earliest dominant infiltrating population in early AKI, initiating inflammatory responses [31].

Complementing these findings, single-cell analysis of crystal nephropathy (CN) demonstrated that KIM-1^+^ injured proximal tubules adopt a pro-inflammatory secretory phenotype that activates resident macrophages via the secreted phosphoprotein 1 (SPP1)–CD44 axis and subsequently amplifies monocyte recruitment through the CCL2–CCR2 pathway [35]. Blocking integrin α_v_β_6_ markedly suppresses the transcription of inflammatory chemokines, especially CCL2, limiting macrophage infiltration and reducing fibrotic lesion size [40]. In nephrotoxic conditions, CCR2^+^/CX3CR1^+^ monocytes are preferentially recruited to the glomerulus, where they persist within glomerular capillaries and contribute to sustained inflammation [41].

### 2.2. Mechanosensitive Infiltration and Differentiation

Recent studies identified Piezo1, mechanosensitive ion channels, as critical regulators of macrophage infiltration. Piezo1 senses increased mechanical stress within fibrotic kidneys, promoting Ca^2+^ influx and activating downstream Calpain and Notch signaling. This, in turn, enhances the expression of CCL2 and CCR2, facilitating macrophage recruitment and activation. Conditional deletion of myeloid Piezo1 markedly reduced macrophage infiltration and fibrosis, highlighting the importance of mechanical stress as a chemoattractant signal in fibrotic progression [42].

Following recruitment, monocytes infiltrate the renal interstitium and undergo functional specialization, a process tightly regulated by the inflammatory microenvironment. This differentiation begins even within the inflamed vasculature, where monocytes start expressing macrophage markers under the influence of CCR2 and endothelial tumor-necrosis factor receptor 2 (TNFR2) signaling, establishing a pro-inflammatory feed-forward loop that maintains macrophage infiltration [41]. Macrophage motility is also influenced by structural and cytoskeletal regulators; deletion of Tead1 (a downstream effector of TAZ) reduces macrophage proliferation and migration [43], while the activation of the YAP signaling pathway by tubular integrin α_v_β_6_ enhances chemotaxis and pro-inflammatory differentiation via increased secretion of the macrophage growth factor IL-34 [40]. Furthermore, macrophage motility depends on the Src family kinase HCK; HCK inhibition reduces M1-associated inflammatory gene expression [44].

### 2.3. Local Proliferation and Survival of Macrophages

The localized proliferation of macrophages within the kidney significantly contributes to their overall accumulation, amplifying the inflammatory and fibrotic response. This expansion is primarily driven by macrophage-colony stimulating factor (M-CSF) signaling via its receptor c-fms (CSF-1R). Targeting this axis with anti-c-fms antibodies markedly decreases macrophage proliferation and accumulation in renal allograft models, reducing interstitial infiltration, tubular injury, and tubulitis, without affecting circulating monocyte or T-lymphocyte accumulation [20,45,46].

In addition to CSF-1R–dependent pathways, recent single-cell and spatial transcriptomic studies show that the expansion of proliferative cycling macrophages (Mki67^+^Top2a^+^) in fibrotic kidneys is further driven by platelet-derived cues acting through platelet-derived growth factor-β (PDGFβ)–PDGFRβ and SPP1–CD44 signaling [34].

IL-34 produced by tubular epithelial cells (TECs) has been shown to act through c-FMS and PTP-ζ to promote macrophage survival and local proliferation within the injured kidney, exacerbating acute injury and accelerating fibrosis progression. Even in the presence of CSF-1, IL-34 exerts a nonredundant role, highlighting its importance as a paracrine driver of macrophage accumulation [47].

## 3. Phenotypic Spectrum of Macrophages in Renal Fibrosis

### 3.1. Pro-Inflammatory Macrophages and Early Injury

Pro-inflammatory M1 macrophages play a detrimental role during the early phase of AKI and drive the transition toward kidney fibrosis [18,48]. Following tissue injury, circulating monocytes are rapidly recruited to the glomerulus and tubulointerstitium, where they differentiate into M1 macrophages. These cells initiate Th1-type immune responses, releasing cytotoxic and inflammatory mediators that exacerbate tissue injury. M1 macrophages are characterized by high expression of induced nitric oxide synthase (iNOS), major histocompatibility complex (MHC) class II, and CD86, and they secrete pro-inflammatory cytokines such as IL-1β, IL-6, IL-12, and TNF-α, along with chemokines like CXCL1, monocyte chemoattractant protein 1 (MCP-1), and reactive species, including reactive oxygen species (ROS) and nitric oxide (NO) [19,49,50]. In early renal inflammation, the accumulation of M1 macrophages correlates strongly with tissue destruction, and their depletion at this stage significantly mitigates kidney injury and preserves renal function [18].

The sustained activation of M1 macrophages in fibrosis is regulated by specific intracellular signaling pathways. The Toll-like receptor 4 (TLR4)/NF-κB axis, often coupled with the pattern-recognition receptor macrophage-inducible C-type lectin (Mincle), is a central pathway maintaining the inflammatory phenotype. Mincle is selectively expressed in M1 macrophages, and its transcription is directly driven by TLR4-NF-κB signaling. Through the Syk pathway, Mincle amplifies the production of M1-associated mediators, sustaining pro-inflammatory activation. Pharmacological inhibition or genetic ablation of Mincle or Syk markedly suppresses these inflammatory mediators and attenuates renal inflammation [49]. In parallel, endoplasmic reticulum (ER) stress contributes to persistent M1 activation through the IRE1α/XBP1s–p38 MAPK pathway. Inhibiting ER stress reduces M1 polarization and significantly alleviates renal injury and fibrosis [50].

Beyond canonical TLR4–NF-κB signaling, the junctional adhesion molecule-like protein (JAML), a macrophage-specific molecule, has recently been implicated in sustaining M1 polarization. JAML promotes macrophage activation through the Mincle–Syk signaling cascade, thereby enhancing pro-inflammatory cytokine production while suppressing efferocytosis [51].

### 3.2. Pro-Fibrotic Macrophages and Tissue Remodeling

Macrophages are critically involved in the development of kidney fibrosis, a hallmark of progressive renal disease (Table 2). The macrophage population implicated in fibrosis is heterogeneous, with the F4/80^hi^ resident subset playing a pathogenic role in chronic injury models. In the repeated low-dose cisplatin (RLDC) model, depletion of F4/80^hi^ macrophages markedly alleviated renal fibrosis, reducing collagen deposition and myofibroblast accumulation, along with a decrease in CD206^+^ M2 macrophages. M2 macrophages contribute to fibrosis by supplying pro-fibrogenic growth factors and matrix metalloproteinases (MMPs) [52]. In vivo, MMP-9 co-localizes with α-smooth muscle actin (α-SMA)-positive myofibroblasts in the unilateral ureteral obstruction (UUO) model, supporting its role in myofibroblast generation [53]. Macrophage MMP-9 also contributes by cleaving SPP1, generating a fragment that enhances the recruitment of more macrophages, which exacerbates the fibrotic process [54].

Single-cell and spatial transcriptomic profiling have revealed multiple disease-specific macrophage states, broadening our understanding of macrophage-driven fibrotic programs. In a study using the reversible UUO (R-UUO)-based AKI–CKD transition model, researchers reported that monocytes expand upon arrival in the injured tissue, expressing ECM-modifying genes such as *TGFB1*, *THBS1*, *FN1*, and *TG2.* These cells then transition into CCR2^+^ macrophages, which maintain SPP1-dependent fibroblast activation, thereby stabilizing the developing fibrotic niche [30]. In diabetic kidney disease (DKD), a distinct TGF-β^+^Arginase 1^+^ (ARG1^+^) macrophage population promotes mesangial remodeling by inducing the conversion of mesangial cells into α-SMA^+^ myofibroblasts, contributing to mesangial matrix expansion [33]. In lupus nephritis (LN), spatial transcriptomic analyses further identified C1qa^+^AIF1^+^ inflammatory macrophages enriched in tubulointerstitial regions, where they produce profibrotic mediators including TGF-β, PDGFβ, CCL2, and SPP1. The same chemokines are also released by injured tubules, enhancing macrophage recruitment and creating a self-reinforcing inflammatory–fibrotic microenvironment [36]. Recent AKI-derived single-cell studies also describe a CCL6^+^CCR2^+^ARG1^+^ macrophage subset originating from circulating CCR2^+^ monocytes. This population expresses CCL6, CCL9, TGF-β, and Lgals3, and exhibits strong engagement of fibrosis-related ligand–receptor pathways [37]. However, genetic ablation of infiltrating macrophages by CCR2 knockout (KO) had minimal impact on fibrotic progression [54].

A subset of SPP1^+^ macrophages, instructed by platelet-derived CXCL4, secretes pro-fibrotic mediators such as TGF-β, fibronectin, and semaphorin 3 (Sema3) that directly activate myofibroblasts. The CXCL4–SPP1^+^ macrophage axis thus represents a platelet-immune-stromal signaling circuit that perpetuates fibrosis through reciprocal activation between macrophages, platelets, and stromal cells [55]. Human CKD single-cell analyses similarly revealed an expanded SPP1^+^ macrophage population that accumulates in fibrotic regions and is actively recruited by chemokine-secreting injured tubules, reinforcing a self-sustaining fibrosis loop [32]. In this context, recent single-cell transcriptomic studies demonstrated that monocytes and resident macrophages, consistently observed in both murine and human lupus nephritis, constitute a conserved injury-associated myeloid (IAM) program. This program is characterized by high expression of SPP1, TREM2, APOE, lysosomal genes, and cholesterol-handling pathways. These SPP1^+^ myeloid cells localize within fibrotic interstitial regions and represent a continuum in which infiltrating monocytes progressively acquire macrophage-like phenotypes during injury and subsequently accumulate as resident populations as chronicity increases. Functionally, SPP1^+^ myeloid cells integrate inflammatory and metabolic cues to regulate apoptotic cell clearance, lipid processing, and matrix remodeling, thereby linking acute injury responses to sustained fibroblast activation. Their increased abundance correlates with higher chronicity indices, which reflect tubulointerstitial fibrosis and glomerular scarring. Based on studies in kidney, persistent secretion of SPP1 and related mediators by these IAM cells has been proposed to potentiate TGF-β–dependent signaling and mechanotransduction pathways in surrounding stromal cells, thereby promoting ECM deposition and maintenance of a fibrotic niche [56].

Recent studies have suggested mechanistic roles for M2 macrophages in kidney fibrosis. Following kidney injury, both infiltrating and resident macrophages upregulate coagulation factors such as F13A1 in a Ca^2+^-dependent manner, leading to fibrin matrix stabilization and enhanced fibrotic remodeling [58]. Also, macrophage-derived Wnt ligands (including Wnt1, Wnt3a, Wnt5a, and Wnt7a) have been identified as potent paracrine factors that activate β-catenin signaling in neighboring fibroblasts. Conditional deletion of the Wnt transporter Wntless (Wls) in myeloid cells attenuates fibroblast activation, ECM accumulation, and collagen deposition in UUO models, underscoring the indispensable role of macrophage-derived Wnt signaling in fibrogenesis [57]. Also, M2 macrophages further enhance ECM stiffness by secreting TGF-β and PDGF, which stimulate fibroblast LOX expression and collagen crosslinking, thereby intensifying mechanotransduction within the fibrotic niche [59,60,61].

## 4. Regulatory Layers Shaping Pro-Fibrotic Macrophage States

**Figure 1:**
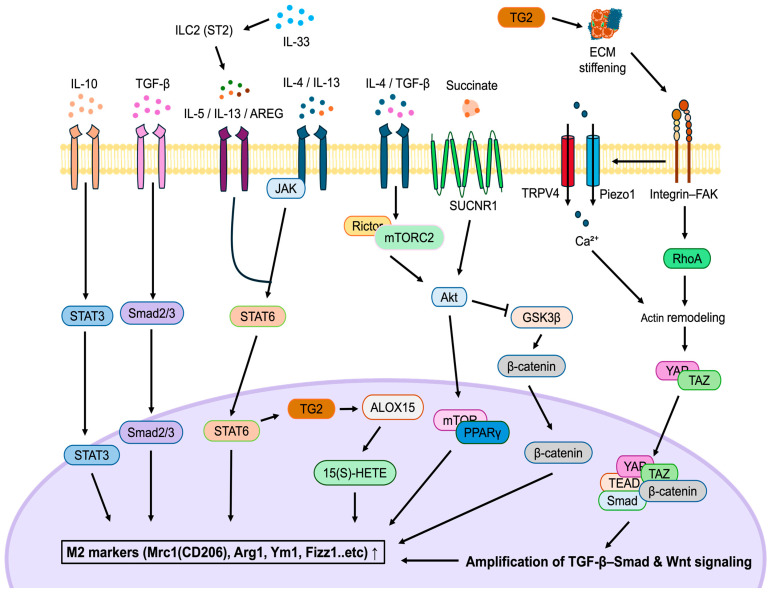
Cytokine- and mechanotransduction-driven programming of pro-fibrotic M2 macrophages in kidney fibrosis. This figure illustrates the major cytokine, metabolic, and mechanical signaling pathways that drive M2 macrophage polarization and stabilize pro-fibrotic phenotypes in the injured kidney. Th2 cytokines IL-4 and IL-13 initiate classical M2 programming by activating the JAK–STAT6 pathway, inducing transcription factors such as IRF4 and KLF4, and promoting the expression of M2 markers including ARG1, FIZZ1, YM1, and MRC1. STAT6 signaling also upregulates transglutaminase 2 (TG2), which enhances ALOX15 expression and 15(S)-HETE production and increases ECM stiffness, further reinforcing M2 activation. Persistent IL-10 and TGF-β feedback loops prolong STAT6 activity, converting an initially reparative response into chronic pro-fibrotic signaling. Epithelial-derived IL-33 additionally amplifies the Th2 cytokine milieu by activating ILC2s through the ST2 receptor, increasing IL-5, IL-13, and AREG production and promoting sustained M2 polarization. Meanwhile, TGF-β–Smad2/3 and IL-10–STAT3 pathways suppress pro-inflammatory programs and stimulate fibroblast activation, establishing a self-reinforcing IL-10– and TGF-β–rich microenvironment. Integrin–FAK–RhoA signaling and mechanosensitive channels such as Piezo1 and TRPV4 increase actomyosin tension and Ca^2+^ influx, driving YAP/TAZ nuclear translocation. Nuclear YAP/TAZ interacts with TEAD, Smad, and β-catenin to amplify TGF-β–Smad and Wnt pathways, enhancing M2 gene expression and sustaining fibrotic programming.

Sustained activation of M2 macrophages can exacerbate fibrosis by stimulating fibroblast proliferation and promoting ECM deposition [17]. The polarization and functional specialization of M2 macrophages are intricately orchestrated by a multilayered regulatory network involving cytokine-mediated signaling, metabolic and mechanical crosstalk, and epigenetic programming [23]. This sophisticated regulation not only determines the initiation of M2 differentiation but also stabilizes the fibrotic macrophage phenotype under chronic injury (Figure 1).

### 4.1. Cytokine-Mediated Regulation of M2 Programming

Within the fibrotic kidney microenvironment, cytokines are pivotal in determining macrophage fate and functional reprogramming of macrophages. Following inflammatory injury, various cytokines are secreted simultaneously, acting not only as inflammation regulators but also as key determinants that induce macrophage polarization and modulate fibrotic progression [62]. Importantly, the composition of this cytokine milieu is also shaped by stromal signaling pathways; notably, stromal STAT3 activity enhances pro-inflammatory cytokine production, whereas its loss shifts the environment toward IL-10– and TGF-β–enriched conditions that favor M2-associated programs [63]. M2 polarization begins when resting macrophages (M0) or previously activated M1 macrophages sense these cytokine cues within the injured renal milieu. Each cytokine activates a specific intracellular cascade, the divergence of which gives rise to functionally distinct M2 subsets [23,39].

The prototypical axis driving M2 polarization is initiated by the Th2 cytokines IL-4 and IL-13. Upon receptor binding, these cytokines activate the JAK-STAT6 pathway, leading to nuclear translocation of STAT6 and induction of critical transcription factors, including IRF4 and KLF4. These transcriptional regulators cooperatively upregulate canonical M2 signature genes (e.g., *ARG1*, *FIZZ1*, *YM1*, and *MRC1*), thereby establishing the M2 phenotype, which is specialized for tissue remodeling and anti-inflammatory functions [15,25]. While IL-4/IL-13–STAT6 signaling defines classical M2 polarization, recent evidence indicates that in the fibrotic kidney, this pathway becomes self-sustaining rather than transient. Persistent STAT6 activation can be maintained through autocrine IL-10 and TGF-β feedback loops, which amplify ECM production and fibroblast activation [17,39]. Thus, the same cytokine signaling axis that promotes inflammation resolution after acute injury paradoxically drives pathological fibrosis when chronically sustained. Furthermore, IL-4 stimulation induces transglutaminase 2 (TG2) expression via the JAK-STAT6 axis, and intracellular TG2 activity is required for the upregulation of ALOX15, a lipoxygenase whose metabolite, 15(S)-HETE, further amplifies M2 programming. This IL-4–STAT6–TG2–ALOX15 signaling cascade integrates mechanical and cytokine-mediated cues, enabling TG2 to simultaneously remodel the ECM and reinforce M2 macrophage activation [64].

Notably, this classical IL-4/IL-13–STAT6 axis is further reinforced by upstream epithelial-derived cues. Among these, the alarmin cytokine IL-33, released from damaged tubular cells, engages the suppression of tumorigenicity 2 (ST2) receptor on group 2 innate lymphoid cells (ILC2s), prompting the production of IL-5, IL-13, and amphiregulin (AREG). Through this mechanism, IL-33 amplifies the Th2 cytokine milieu, enhancing both M2 polarization and tissue repair. When IL-33 signaling persists in chronic injury, the resulting prolonged M2 activation and continuous TGF-β output establish a feed-forward loop that accelerates fibrosis [65].

Alternatively, TGF-β and IL-10 also promote macrophage polarization toward a fibrogenic M2 phenotype through distinct intracellular routes. TGF-β activates the Smad2/3 signaling pathway, whereas IL-10 engages STAT3. Both pathways suppress pro-inflammatory activity while inducing fibrotic mediators such as collagen I and α-smooth muscle actin (α-SMA) in neighboring fibroblasts [15,17,25]. These macrophages, in turn, secrete high levels of IL-10 and TGF-β, forming a self-reinforcing circuit that stabilizes their alternative activation and perpetuates the pro-fibrotic microenvironment [39,66].

### 4.2. Mechanotransduction-Mediated Regulation of M2 Programming

Mechanical cues generated by progressive ECM stiffening function synergistically with metabolic and cytokine-derived signals to potentiate M2 macrophage programming [43]. In the fibrotic niche, increased ECM stiffness is sensed by macrophages through their mechanosensing machinery, which activates the Integrin–FAK–RhoA pathway and induces actin cytoskeleton rearrangement. Beyond this canonical integrin-dependent pathway, stiff matrices also promote integrin clustering and ROCK-mediated contractility. Concurrently, mechanosensitive channels like Piezo1 and TRPV4 induce rapid Ca^2+^ influx, further elevating cytoskeletal tension and reinforcing downstream nuclear signaling [60,67,68]. This mechanical activation suppresses the Hippo signaling pathway, leading to the nuclear translocation of the normally restrained YAP/TAZ transcriptional co-activators [69]. Once in the nucleus, YAP/TAZ form complexes with TEAD, Smad, and β-catenin, thereby functioning as co-regulators that amplify TGF-β–Smad and Wnt signaling [43,59]. Studies have demonstrated that simultaneous stimulation with TGF-β and Wnt5a enhances YAP/TAZ nuclear accumulation, which markedly upregulates M2-related genes (e.g., *ARG1*, *YM1*). In contrast, pharmacological inhibition using Verteporfin (VP) or macrophage-specific deletion of *Taz* (*Csf1r-Cre*; *Taz^fl^/^fl^*) suppressed both M2 polarization and fibrotic responses. These findings indicate that the mechanical cues from a stiffened ECM do not merely accompany fibrotic signaling but actively cooperate with growth factor pathways to sustain macrophage M2 polarization. This mechanochemical feedback loop drives the fibrotic milieu, establishing a self-reinforcing circuit that contributes to fibrosis progression [43,70,71]. Importantly, sustained exposure to stiff ECM can imprint a form of “mechanical memory” in macrophages, whereby chromatin remodeling and persistent YAP/TAZ activity stabilize a pro-fibrotic transcriptional program even after mechanical inputs diminish [60,72].

### 4.3. Epigenetic and Post-Transcriptional Regulation of M2 Programming

Once polarized, the macrophage phenotype is not a fixed state but remains dynamically modulated by intracellular and extracellular mechanisms, including epigenetic and post-transcriptional regulation (Table 3). These processes play a pivotal role in shaping the trajectory through which M2 macrophages transition toward a pro-fibrotic phenotype under chronic injury conditions. In this regard, epigenetic and post-transcriptional mechanisms constitute the molecular foundation of macrophage memory and plasticity, enabling the fine-tuning of their functional identity over time [73].

Non-coding RNAs have emerged as central regulators of macrophage fate determination. In sepsis-induced AKI models, the long non-coding RNA (lncRNA) NEAT1 has been implicated in modulating inflammatory responses. NEAT1 functions as a molecular sponge for miR-125a-5p, thereby suppressing its activity. Inhibition of NEAT1 releases miR-125a-5p, which subsequently downregulates its target TRAF6, leading to suppression of the TRAF6/TAK1 signaling cascade. This attenuation promotes M2 polarization and contributes to inflammatory resolution, suggesting that NEAT1 acts as a regulatory hub linking non-coding RNA networks with macrophage activation pathways [74].

Multiple microRNAs (miRNAs) also exert direct control over macrophage polarization during renal fibrosis. In a folic acid–induced kidney fibrosis model, miR-150 expression was markedly elevated, whereas administration of an antisense inhibitor (LNA-anti-miR-150) attenuated both M1 and M2 polarization, ultimately alleviating interstitial fibrosis [75]. More recently, the circular RNA circACTR2 was shown to be highly expressed in M2 macrophages, functioning as a sponge for miR-200c. Since miR-200c suppresses YAP signaling, circACTR2-mediated sequestration of miR-200c results in YAP activation and enhanced M2 polarization, highlighting a novel post-transcriptional circuit that links circular RNA and mechanotransductive pathways [76].

Beyond sponge effects, individual miRNAs can directly modulate intracellular signaling cascades to control macrophage fate. In an aristolochic acid–induced renal fibrosis model, studies demonstrated that miR-382 was specifically upregulated in macrophages from fibrotic kidneys. Elevated miR-382 targeted the mRNA of the signaling regulator SIRP-α, suppressing its expression and leading to sustained activation of STAT3, thereby promoting M2-like polarization. This finding provides mechanistic insight into how discrete miRNAs fine-tune intracellular signaling to dictate macrophage functional outcomes [77].

**Table 3:** Epigenetic and Post-Transcriptional Regulators of Pro-Fibrotic Macrophage Programming. Abbreviation: AKT (Protein kinase B [PKB]), STAT3 (Signal transducer and activator of transcription 3), circACTR2 (Circular RNA derived from ACTR2), CD206 (Mannose receptor C-type 1), EZH2 (Enhancer of zeste homolog 2), FAO (Fatty acid oxidation), H3K27me3 (Histone H3 Lysine 27 Trimethylation), IRF4 (Interferon regulatory factor 4), JMJD3 (Jumonji domain-containing protein 3), NEAT1 (Nuclear paraspeckle assembly transcript 1), NF-κB (Nuclear factor kappa-light-chain-enhancer of activated B cells), SIRT6 (Sirtuin 6), SIRP-α (Signal-Regulatory Protein-α), STAT3 (Signal transducer and activator of transcription 3), TRAF6 (TNF receptor-associated factor 6), PTEN (Phosphatase and tensin homolog), TRAF6 (Tumor Necrosis Factor Receptor-Associated Factor 6), TAK1 (Transforming Growth Factor-beta-Activated Kinase 1), YAP (Yes-associated protein), TAZ (Transcriptional co-activator with PDZ-binding motif).

Category	Regulatory	Mechanism/Target	Effect	Impact on Fibrosis	Reference
Long non-coding RNAs (lncRNAs)	NEAT1	Sponges miR-125a-5p → releases TRAF6	Suppresses TRAF6/TAK1 pathway	Facilitates inflammatory resolution (AKI) but may contribute to chronic M2 bias	[74]
Circular RNAs (circRNAs)	circACTR2	Sponges miR-200c → derepresses YAP	Enhances YAP/TAZ signaling	Enhances M2 pro-fibrotic signaling	[76]
MicroRNAs (miRNAs)	miR-150	Broad suppression of M1/M2 polarization programs	Reduces macrophage activation	Attenuates interstitial fibrosis	[75]
	miR-382	Targets SIRP-α	Sustains STAT3 activation	Promotes progression of renal fibrosis	[77]
Histone deacetylases	SIRT6	Deacetylates STAT6	Suppresses NF-κB; enhances FAO	Supports repair acutely, but persistent signaling fosters chronic fibrosis	[78]
Histone demethylases	JMJD3	Removes H3K27me3 at IRF4 promoter	Enhances IRF4 transcription ↑	Increases CD206^+^ M2 accumulation and collagen deposition	[79]
Histone methyltransferases	EZH2	Deposits H3K27me3 at PTEN promoter	PI3K–AKT/STAT3 disinhibition	Accelerates fibrosis through PI3K/Akt-STAT3 pathways	[80]

Epigenetic modifiers that directly alter chromatin structure also play a decisive role in M2 macrophage regulation [73]. SIRT6, a NAD^+^-dependent deacetylase and metabolic epigenetic regulator, modulates macrophage polarization through STAT6 deacetylation and inhibition of NF-κB signaling. SIRT6 promotes M2-associated FAO and autophagy, facilitating tissue repair in acute injury. However, its persistent activation contributes to fibrosis, primarily through sustained TGF-β/Smad3 and Wnt/β-catenin signaling [78].

Additionally, the histone demethylase Jumonji domain-containing protein-3 (JMJD3) acts downstream of IL-4/STAT6 signaling, removing the repressive histone mark H3K27me3 at the promoter region of *IRF4*, a master transcription factor for M2 programming. This demethylation facilitates *IRF4* transcription and stabilizes the M2 phenotype. In UUO-induced kidney fibrosis, inhibition of JMJD3 with GSK-J4 reduced CD206^+^ M2 macrophage accumulation and collagen deposition, confirming its profibrotic role [79]. Illustrating the complexity of this epigenetic network, the methyltransferase Enhancer of zeste homolog 2 (EZH2) also promotes M2 polarization, despite having the opposite enzymatic function. Whereas JMJD3 removes the repressive H3K27me3 mark to activate the M2-promoting factor IRF4, EZH2 deposits this same mark to suppress a key signaling inhibitor, the tumor suppressor *PTEN*. In ischemia–reperfusion injury (IRI) and folic acid-induced nephropathy models, EZH2-mediated suppression of *PTEN* led to aberrant activation of PI3K/Akt and STAT3 signaling, which in turn enhanced M2 polarization and exacerbated fibrosis progression [80].

In addition, recent cross-species STAT3 interactome analyses have identified P300 as a conserved STAT3-binding partner and influential epigenetic co-activator in fibrotic signaling. P300-mediated acetylation of STAT3 at Lys685 increases H3K27ac and strengthens STAT3-dependent transcriptional programs associated with proliferation and matrix remodeling. Inhibition of P300 disrupts STAT3 acetylation and reduces fibrogenic gene expression, suggesting that STAT3-linked chromatin modifiers serve as key amplifiers of fibrotic transcriptional circuits. Given STAT3’s position downstream of IL-10, TGF-β, and PI3K/Akt in macrophages, similar P300–STAT3–H3K27ac modules may operate in macrophages to sustain pro-fibrotic activation during chronic kidney injury [81].

### 4.4. Metabolic and Mitochondrial Regulation of Macrophage Programming

The metabolic reprogramming of macrophages is a key driver of their pathological activity in renal fibrosis. This functional plasticity, which dictates whether the macrophage contributes to acute injury or chronic progression, is governed by a deliberate reprogramming of core energy systems. Distinct metabolic states are precisely regulated by transcription factors, integrating upstream signaling with downstream metabolic output to fuel specialized functions (Table 4).

#### 4.4.1. Metabolic Characteristics of M1-Type Macrophages

**Glycolysis**

Classically activated M1 macrophages undergo a pronounced shift from oxidative phosphorylation (OXPHOS) to aerobic glycolysis upon stimulation with PAMPs and endogenous danger signals, typically through TLR4 and STAT1 signaling [82,83]. This switch is orchestrated by hypoxia-inducible factor-1α (HIF-1α), a transcription factor stabilized even under normal (normoxic) conditions via both the TLR/NF-κB and AKT/mTOR pathways, which upregulate key glycolytic enzymes such as hexokinase 2 (HK2), 6-phosphofructo-2-kinase/fructose-2,6-bisphosphophatase 3 (PFKFB3), pyruvate kinase M2 (PKM2), and lactate dehydrogenase A (LDHA) [82,83,84]. The glycolytic switch enhances ATP production speed, supplies intermediates for the pentose phosphate pathway (PPP), and generates nicotinamide adenine dinucleotide phosphate (NADPH), supporting ROS and NO production critical for pathogen killing [83]. HIF-1α–driven glycolysis is directly associated with the expression of pro-inflammatory cytokines, including IL-1β, IL-6, and TNF-α, while inhibition of glycolysis with 2-deoxyglucose (2-DG) markedly reduces cytokine output and phagocytic capacity [85]. Moreover, the TCA intermediate succinate accumulates as a consequence of disrupted flux; this metabolite stabilizes HIF-1α and drives IL-1β transcription, thereby creating a self-sustaining inflammatory loop [82]. Conversely, blocking PFKFB3 or PKM2 activity diminishes pro-inflammatory cytokines and dampens macrophage effector functions, demonstrating their critical role as metabolic checkpoints [86,87].

In renal fibrosis, several studies highlight that macrophage glycolysis fuels inflammation and extracellular matrix remodeling within injured kidneys. A recent study demonstrated that activation of the glycolytic enzyme PFKFB3 in myeloid cells exacerbates renal fibrosis in the UUO model [87]. Deletion of *PFKFB3* in macrophages (*PFKFB3* ∆Mϕ) reduced macrophage infiltration, M1/M2 abundance, and myofibroblast transition, thereby attenuating fibrosis [87]. The metabolites derived from PFKFB3 stabilize HIF-1α, altering macrophage phenotype, and enhancing TGF-β-dependent ECM synthesis. This defines a PFKFB3–HIF-1α–TGF-β axis central to fibrotic signaling driven by macrophages. Another study illustrated that TREM2-dependent PI3K–AKT–mTOR signaling sustains macrophage viability and metabolic activation. Loss of *Trem2* compromises mTOR phosphorylation, induces apoptosis, and exaggerates M1 skewing via compensatory JAK–STAT activation, worsening fibrosis in both UUO and IRI models [86].

Myeloid-specific *Hif-1α* deletion attenuates fibrosis in both UUO and IRI models [88]. Maladaptively repaired TECs secrete cytokines and exosomes, activating NF-κB–HIF-1α–glycolysis signaling in macrophages. NF-κB directly binds the *HIF1α* promoter (via p65 occupancy verified by ChIP-qPCR), establishing transcriptional control over glycolytic enzymes (HK2, GLUT1) [88]. Inhibition of NF-κB with BAY-11-7082 or blockade of glycolysis with 2-DG suppressed lactate accumulation and reduced α-SMA, collagen-1, and fibronectin expression in fibrotic kidneys. Thus, HIF-1α serves as both a metabolic effector and a redox-responsive transcriptional node maintaining a pro-inflammatory macrophage phenotype during maladaptive repair [88].

**PPP**

The PPP constitutes a vital metabolic branch parallel to glycolysis, generating NADPH via the oxidative enzyme glucose-6-phosphate dehydrogenase (G6PD). In macrophages, NADPH serves dual physiological roles. It sustains ROS and NO production for host defense, and it provides a reducing equivalent for glutathione recycling and redox balance. Under renal injury, sepsis, or hypoxic conditions, PPP upregulation enhances NADPH flux, which drives NOX2-mediated ROS generation and perpetuates oxidative inflammation [88,89]. In renal fibrosis, the redox role of NADPH becomes context-dependent. It promotes pathogen clearance and macrophage survival during acute injury; however, when chronic NADPH excess sustains oxidative signaling, it stabilizes TGF-β and SMAD3 activity and induces collagen I/III transcription. Changes in the PPP activity drive the evolution of macrophages from a pro-inflammatory (M1) state to a pro-fibrotic hybrid state. This transition from adaptive to maladaptive PPP function is crucial for making fibrosis a chronic, persistent disease [27].

G6PD, the rate-limiting enzyme of PPP, is consistently upregulated in both experimental and clinical models of kidney injury and fibrosis. LPS or ischemic stimulation activates the TLR4–NF-κB–G6PD axis, boosting NADPH and ROS production. In macrophages derived from UUO and IRI kidneys, G6PD overexpression amplified IL-1β, TNF-α, and MCP-1 secretion, whereas G6PD inhibition or knockdown reduced tubulointerstitial macrophage activation and improved renal function [88,90,91].

#### 4.4.2. Metabolic Characteristics of M2-Type Macrophage

**TCA cycle**

M2 macrophages maintain an intact TCA cycle, in contrast to the truncated configuration observed in glycolytic M1 macrophages [82]. Activated STAT6 drives transcription of peroxisome proliferator-activated receptor γ (PPARγ) and its co-activator PPARγ co-activator 1 β (PGC-1β), initiating a coordinated upregulation of mitochondrial biogenesis and FAO enzymes such as CPT1A, ACADL, and HADHA. These transcriptional events provide the acetyl-CoA substrates that fuel the TCA cycle and sustain OXPHOS-coupled ATP generation [92,93,94].

**OXPHOS and FAO**

OXPHOS is suppressed in pro-inflammatory macrophages due to increased activity of STAT1. In contrast, the metabolic shift provides the crucial metabolic foundation for STAT6-dependent polarization toward the M2 phenotype reprogramming, driven by STAT6, which enhances OXPHOS and FAO [93,99]. This metabolic shift provides the crucial metabolic foundation for STAT6-dependent polarization toward the M2 phenotype [100]. STAT6 activation drives the expression of the transcription factor PPARγ and its coactivator PGC-1β. This complex subsequently promotes the transcription of genes related to FAO and enhances both mitochondrial biogenesis and function [97,98]. The resulting increase in FAO flux provides an abundant supply of acetyl-CoA substrates for OXPHOS, which in turn drives efficient ATP synthesis. Since M2 macrophages rely on efficient OXPHOS with lower electron leaks and do not accumulate significant amounts of succinate, their ROS levels are significantly lower than those of M1 macrophages [95,96].

#### 4.4.3. Metabolic–Immune Signaling–Mediated Regulation of M2 Programming

M2 macrophage polarization in renal fibrosis is further shaped by metabolic–immune signaling nodes that integrate environmental cues with intracellular energy status. By integrating these cues, macrophages undergo continuous functional reprogramming, dynamically adapting their phenotype to the evolving tissue milieu [101,102].

IL-4-mediated signaling operates in concert with the JAK–STAT6 axis to activate the PI3K–Akt pathway, which regulates cellular metabolism and survival [25]. Recent evidence has demonstrated that this pathway is modulated upstream by the mTORC2 complex, which provides a metabolic foundation that reinforces M2 polarization. In UUO and IRI models, studies have reported a marked increase in Rictor-dependent mTORC2 activation within fibrotic renal macrophages. Conditional deletion of Rictor in macrophages (*Csf1r-Cre*; *Rictor^fl^/^fl^*) significantly reduced Akt phosphorylation at Ser473, leading to decreased expression of M2-associated genes (*ARG1*, *MRC1*), accompanied by attenuated collagen deposition. These findings suggest that the mTORC2–Akt axis acts in parallel with JAK–STAT6 signaling to potentiate M2 polarization, driving metabolic reprogramming toward OXPHOS and FAO, thereby sustaining macrophage survival and functional stability [101]. Thus, the mTORC2–Akt pathway serves as a critical integrative hub linking metabolic and immune signaling in the fibrotic niche.

A pathogenic signaling axis linking metabolic stress to profibrotic macrophage programming is mediated by the G-protein–coupled receptor SUCNR1. Activation of SUCNR1 on macrophages initiates a defined intracellular signaling cascade characterized by phosphorylation of Akt at Ser473, subsequent inactivation of GSK3β via Ser9 phosphorylation, and stabilization of non-phosphorylated β-catenin. This β-catenin accumulation promotes its nuclear translocation and transcriptional induction of CTGF, a central mediator of macrophage–fibroblast communication. Through this signaling module, SUCNR1 engagement reprograms macrophages toward a pro-fibrotic M2-like phenotype, reflected by increased expression of *ARG1*, *FIZZ1/CHIL3*, *MGL2*, and *IL10*, along with elevated secretion of profibrotic factors including galectin-3, MMP9/12/13, PDGF, and CTGF. Conditioned medium derived from SUCNR1-activated macrophages is sufficient to stimulate fibroblast proliferation and myofibroblast differentiation, whereas SUCNR1 knockdown or CTGF neutralization abrogates these paracrine responses. In vivo, depletion of macrophages markedly attenuates fibrosis induced under chronic metabolic stress, demonstrating that the SUCNR1–Akt–GSK3β–β-catenin signaling axis constitutes the core pathway that integrates metabolic dysregulation with macrophage-driven fibroblast activation during renal fibrogenesis [102].

### 4.5. Cell-Stress and Autophagy-Mediated Regulation of Macrophage Programming

#### 4.5.1. Mitochondrial Dysfunction and Impaired Mitophagy

The kidney requires a substantial amount of mitochondria because its vital functions of filtering waste from the blood and maintaining fluid and electrolyte homeostasis are extremely energy-intensive [103]. Mitochondria also play a pivotal role in macrophage function by regulating energy metabolism, redox balance, and cell signaling essential for immune responses [104]. Chronic inflammation results in mitochondrial dysfunction and promotes macrophage polarization to the M2 phenotype. These M2 macrophages can then transform into pro-fibrotic macrophages by producing TGF-β in the situation of kidney fibrosis [20].

Normally, the cell can remove damaged or abnormal mitochondria through a cellular quality control process called mitophagy [105]. This mechanism prevents the accumulation of dysfunctional mitochondria, thereby averting cellular damage. Mitophagy is initiated by tagging and fragmenting dysfunctional mitochondria in the mitophagosomes. This process is primarily controlled by two regulatory proteins. PINK1 accumulates on damaged mitochondria [106], which then facilitates the recruitment of cytosolic Parkin [107]. Parkin subsequently applies a ubiquitin tag to the outer mitochondrial membrane, marking the mitochondria for degradation [107,108].

In the UUO-induced kidney fibrosis mouse model, the expression level of Parkin is decreased, indicating impaired mitophagy in the fibrotic kidney. TGF-β, which is highly abundant in the fibrotic environment, appears to be a primary driver of this failure, attacking mitochondrial health from two directions. First, TGF-β directly causes mitochondrial dysfunction, as demonstrated by the significantly decreased mitochondrial oxygen consumption and ATP production in macrophages [109]. Second, the fibrotic environment created by TGF-β simultaneously suppresses the mitophagy machinery needed to fix this damage [109]. Likewise, TGF-β exacerbates both mitochondrial dysfunction and mitophagy. This impairment blocks the clearance pathway, resulting in a significant accumulation of dysfunctional mitochondria. Also, mitochondrial dysfunction within renal cells acts as a critical pathological driver by actively engaging the innate immune system. Specifically, the deletion of *COX10* (a Complex IV cofactor) in podocytes results in a failure of OXPHOS and subsequent leakage of mitochondrial DNA (mtDNA) into the cytosol. This release activates the STING pathway, which triggers a robust type I interferon response, subsequently promoting the increased infiltration and persistence of macrophages, thereby fueling the progression of chronic renal inflammation and fibrosis [110].

#### 4.5.2. Dual Role of Macrophage Autophagy in Renal Fibrosis

**Figure 2:**
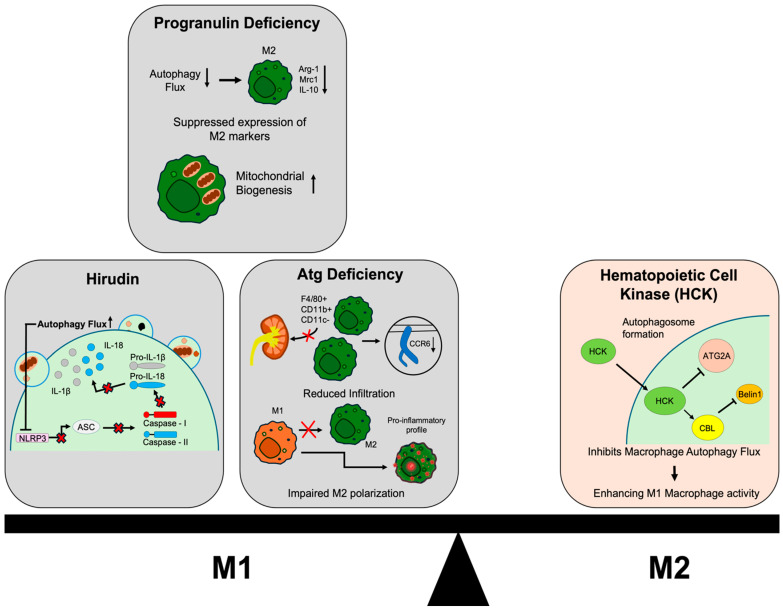
Dual role of macrophage autophagy in renal fibrosis. Autophagy signaling exerts context-dependent effects on fibrotic progression. On the fibrogenic autophagy side, deficiency of ATG5 reduces renal infiltration of F4/80^+^CD11b^+^CD11c^−^ macrophages by downregulating CCR6, thereby decreasing responsiveness to the CCL20 chemotactic signal. ATG5 loss further impairs M2 macrophage polarization, with reduced expression of M2 markers (ARG-1, MRC1, IL-10), shifting macrophages toward a pro-inflammatory phenotype and ultimately attenuating extracellular matrix deposition. Similarly, progranulin deficiency diminishes autophagy flux and mitochondrial homeostasis, suppressing M2-associated gene expression and reducing fibrotic tissue remodeling. Conversely, anti-fibrogenic autophagy acts to limit inflammation-driven fibrosis. Hematopoietic cell kinase (HCK) inhibits macrophage autophagy via interaction with ATG2A and CBL, promoting M1 polarization, proliferation, and migration, and enhancing renal inflammatory infiltration. Deletion or inhibition of HCK restores autophagy, reduces M1-related cytokines, and alleviates fibrosis. In addition, hirudin suppresses renal fibrosis by inducing autophagy and consequently inhibiting activation of the NLRP3/caspase-1–IL-1β/IL-18 inflammasome axis; this protective effect is abolished when autophagy activation is absent.

Recent studies have revealed that macrophage autophagy, including mitophagy, acts as a critical regulator of immune and fibrotic processes in chronic kidney disease. However, the exact function of this pathway is complicated because the type of autophagic flux and the context have a significant impact on whether it drives pathogenesis or promotes regeneration. Therefore, we summarize the evidence demonstrating both the protective and pathogenic roles of autophagy in renal fibrosis progression (Figure 2).

Deficiency of the autophagy-related gene *ATG5* suppresses renal fibrosis in both IRI and UUO models. Following ischemic injury, renal infiltration of F4/80^+^CD11b^+^CD11c^−^ macrophages was markedly reduced in *Atg5* KO mice compared with wild-type controls. Also, CCR6 expression was reduced in *ATG5*-deficient macrophages, resulting in reduced sensitivity to CCL20 chemotactic signals and suppressed renal migration. Moreover, *ATG5* deficiency impairs M2 macrophage polarization, shifting macrophage responses toward a pro-inflammatory profile and resulting in attenuated fibrotic remodeling [111]. Deficient autophagy within renal cells acts as a primary trigger for kidney failure, establishing a direct link between impaired lysosomal degradation and mitochondrial damage. Specifically, the genetic deletion of key autophagy regulators like *ATG5* or *ATG7* in nephrons leads to the accumulation of severely damaged mitochondria in podocytes, which subsequently increases ROS generation and impairs ATP production. This intrinsic metabolic failure drives severe glomerular and tubular pathology, ultimately resulting in focal segmental glomerulosclerosis (FSGS) and organ failure [112]. Also, progranulin, known to play an important role in neuroprotection and regulating lysosomal function, increases autophagy flux in macrophages and promotes M2 polarization. When progranulin is inhibited, the expression of M2 markers such as ARG1, MRC1, and IL-10, along with autophagy flux, is suppressed, thereby suppressing fibrosis and improving mitochondrial biogenesis function [113].

Conversely, appropriate macrophage autophagy often tends to suppress kidney fibrosis. Hematopoietic cell kinase (HCK) inhibits macrophage autophagy in renal fibrosis and inflammation, thereby enhancing M1 macrophage activity and promoting fibrosis. HCK directly inhibits macrophage autophagy flux by interacting with autophagy-related proteins ATG2A and CBL [44]. HCK expression is specifically increased in macrophages in CKD kidneys, and deletion or inhibition of this gene reduces macrophage M1 polarization, proliferation, and migration, resulting in reduced macrophage infiltration in the kidney and suppression of inflammation and fibrosis [100]. Also, Hirudin demonstrates another beneficial aspect of autophagy. Hirudin reduces renal fibrosis by decreasing NLRP3 inflammasome-related protein expression and increasing autophagy-related protein expression. This is because the activation of the NLRP3/caspase-1–IL-1β/IL-18 pathway is inhibited by autophagy induction [114]. These hirudin effects are evident only when autophagy activation is also present.

## 5. Conclusions

Renal fibrosis is characterized by a variety of immune and metabolic changes. Recent studies have shown that macrophages are not passively adapting to the environment but rather act as key regulators of fibrosis. Macrophages infiltrate into damaged renal tissue by the CCR2–CCL2 axis and adapt to the microenvironment and progressively change their transcriptional and metabolic programs [38]. These changes sometimes transform defensive cells that restore tissue homeostasis and clear debris into pathological cells that drive chronic inflammation and fibrosis [37].

The phenotypic transition of macrophages is not a simple or linear process; instead, it is a continuous and dynamic process. Initial inflammatory macrophage activation accelerates tissue damage through the production of cytokines and reactive oxygen species. Over time, sustained exposure to cytokines such as IL-4, IL-13, IL-10, and TGF-β establishes a self-sustaining state that promotes extracellular matrix accumulation and fibroblast activation [15,25,39]. These processes are reinforced by mechanical stress and metabolic alterations, ultimately stabilizing macrophage identity within the fibrotic microenvironment. Signaling pathways such as mTORC2–Akt, TGF-β–Smad, and YAP/TAZ integrate these diverse signals within macrophages to drive a pro-fibrotic phenotype [70,101].

Energy metabolism and mitochondrial quality control are crucial for macrophage function. Inflammatory macrophages rely on glycolysis and the PPP for energy production and redox regulation, whereas fibrotic macrophages maintain OXPHOS and FAO. Impaired autophagy and mitophagy lead to a breakdown in mitochondrial quality control and the accumulation of damaged mitochondria. This increases reactive oxygen species production and enhances TGF-β activity, forming a feedback loop that exacerbates fibrosis. Targeting these metabolic and mitochondrial processes may interrupt this cycle and help restore immune balance in chronic kidney disease. However, macrophage autophagy sometimes plays a dual role in kidney fibrosis. PINK1/Parkin–mediated mitophagy eliminates damaged mitochondria and thereby suppresses fibrosis [109], whereas general macrophage autophagy dependent on Atg5 may instead promote the profibrotic function of macrophages and exacerbate disease [111].

Although there is increasing evidence of metabolic reprogramming and mitochondrial dysfunction, more research is needed to further define the multi-layered control of macrophages in renal fibrosis. It also remains unclear how interactions among macrophage recruitment, polarization, and mechanotransduction coordinate immune–metabolic responses through pathways such as YAP/TAZ, mTORC2, and TGF-β–Smad to shape the fibrotic microenvironment. Furthermore, integrative single-cell, spatial transcriptomic, and metabolic profiling approaches are required to characterize macrophage heterogeneity and identify the critical thresholds that distinguish recovery from irreversible fibrosis, thereby enabling stage-specific and precision anti-fibrotic therapies.

Therapeutic strategies targeting macrophage metabolism and signaling pathways have great potential because they directly address the fundamental regulatory mechanisms of fibrosis. Unlike simple inflammation suppression, these approaches have the advantage of reversing macrophage functions that are locked into a pathological environment. For example, modulating metabolic pathways can restore energy flow and redox balance, thus suppressing ROS overproduction, and the persistence of TGF-β signaling can be suppressed. Modulating mechanosensing signals can mitigate fibrotic responses amplified by ECM stiffening. Restoring autophagy and mitophagy can simultaneously reduce the accumulation of damaged mitochondria, thereby reducing oxidative stress and inflammatory signaling. Strategies that combine immune, metabolic, and mechanosensing signals offer therapeutic potential for fundamentally disrupting macrophage–centered fibrotic feedback loops.

## Figures and Tables

**Table 2:** A list of immune cells involved in different types of kidney disease and their markers with specific functions. Abbreviation: AIF1 (Allograft Inflammatory Factor 1), AKI (Acute kidney injury), ARG1 (Arginase 1), C1qa (Complement component 1q A chain), CCL6 (C-C motif chemokine ligand 6), CCL9 (C-C motif chemokine ligand 9), CKD (Chronic kidney disease), DKD (Diabetic kidney disease), FN1 (Fibronectin 1), F13A1 (Coagulation factor XIII A chain), Lgals3 (Galectin-3), LN (Lupus nephritis), Ly6C (Lymphocyte antigen 6 complex locus C), MMP-9 (Matrix metalloproteinase-9), PDGFβ (Platelet-derived growth factor-β), Sema3 (Semaphorin-3 family), SPP1 (Secreted phosphoprotein 1), TG2 (Transglutaminase 2), TGF-β (Transforming growth factor-beta), THBS1 (Thrombospondin-1), Wnt1 (Wnt family member 1), Wnt3a (Wnt family member 3A), Wnt5a (Wnt family member 5A), Wnt7a (Wnt family member 7A).

Disease Type	Cell Type	Markers/Molecules	Function	Reference
AKI-CKD transition	Monocyte	Ly6C, ARG1, TGF-β, THBS1, FN1, TGM2	Transition into Ccr2^+^ macrophages and maintains Spp1-dependent fibroblast activation	[30]
DKD	Macrophage	TGF-β, ARG1	Promotes mesangial remodeling by inducing their conversion into α-SMA^+^ myofibroblasts	[33]
LN	Macrophage	C1qa, AIF1, TGF-β, PDGFβ, SPP1	Produces profibrotic mediators and activate fibroblasts & myofibroblasts	[36]
AKI	Macrophage	CCL6, CCL9, TGF-β, Lgals3	Engagement of fibrosis-related receptor pathway	[37]
CKD	Macrophage	F4/80	Promotes collagen deposition and myofibroblast accumulation	[52]
		MMP-9	Enhances macrophage recruitment and promotes fibrotic process	[54]
CKD	Macrophage	SPP1, TGF-β, fibronectin, Sema3	Directly activates myofibroblasts	[55]
LN	Myeloid	SPP1	Reinforce ECM deposition to sustain the fibrotic niche	[56]
CKD	Macrophage	Wnt1, Wnt3a, Wnt5a, Wnt7a, Wntless (Wls)	Activates β-catenin signaling in neighboring fibroblasts by Wnt signaling pathway	[57]

**Table 4:** Metabolic Characteristics of Macrophages and Their Outcomes. Abbreviations: ACADL (acyl-CoA dehydrogenase long chain), AKT (AKT Serine/Threonine Kinase), ATP (Adenosine triphosphate), CPT1A (Carnitine palmitoyltransferase 1A), FAO (Fatty acid oxidation), G6PD (Glucose-6-phosphate dehydrogenase), GLUT1 (Glucose transporter type 1), HADHA (Hydroxyacyl-CoA Dehydrogenase Trifunctional Multienzyme Complex Subunit Alpha), HIF-1α (Hypoxia-inducible factor-1 alpha), HK2 (Hexokinase 2), LDHA (Lactate dehydrogenase A), mTOR (Mechanistic Target of Rapamycin), NADPH (Nicotinamide adenine dinucleotide phosphate (reduced)), NF-κB (Nuclear Factor Kappa-light-chain-enhancer of activated B cells), OXPHOS (Oxidative phosphorylation), PFKFB3 (6-phosphofructo-2-kinase/fructose-2,6-bisphosphatase 3), PGC-1β (PPARγ coactivator 1 beta), PI3K (Phosphatidylinositol 3-Kinase), PKM2 (Pyruvate kinase M2), PPARγ (Peroxisome proliferator-activated receptor gamma), PPP (Pentose phosphate pathway), ROS (Reactive oxygen species), STAT1 (Signal Transducer and Activator of Transcription 1), STAT6 (Signal Transducer and Activator of Transcription 6), TCA (Tricarboxylic acid cycle), TGF-β (Transforming Growth Factor Beta), TLR4 (Toll-like Receptor 4).

Macrophage Phenotype	Metabolic Pathway	Key Enzymes/ Regulators	Signaling Pathway	Outcomes	References
M1 (Pro- Inflammatory)	Glycolysis	HK2, PFKFB3, PKM2, LDHA, HIF-1α, GLUT1	TLR4-STAT1, PI3K-AKT-mTOR, PFKFB3-HIF1α-TGF-β, NF-kB-HIF-1α	Enhances ATP production; supplies intermediate for PPP; generates NADPH; supports ROS & NO production	[82,83,84,85,86,87,88]
	PPP	G6PD, NADPH	TLR4-NF-kB-G6PD axis	Sustains ROS and NO production; provides reducing equivalent for glutathione recycling and redox balance; enhances NADPH flux	[88,89,90,91]
M2 (Anti- inflammatory)	TCA cycle	PPARγ	STAT6	Upregulation of mitochondrial biogenesis & FAO enzymes	[92,93,94]
	OXPHOS	PGC-1β	STAT6	Efficient ATP synthesis; low ROS level	[95,96]
	FAO	CPT1A, ACADL, and HADHA	STAT6	Enhances mitochondrial biogenesis and function; provides abundant supply of acetyl CoA for OXPHOS	[92,93,94,97,98]

## Data Availability

No new data were created or analyzed in this study. Data sharing is not applicable to this article.

## Abbreviations

The following abbreviations are used in this manuscript:

2-DG	2-deoxyglucose
AIF1	Allograft inflammatory factor 1 (Iba1)
AKI	Acute kidney injury
α-SMA	Alpha-smooth muscle actin
ARG1	Arginase 1
AREG	Amphiregulin
ATG5	Autophagy-related protein 5
ATG7	Autophagy-related protein 7
C1qa	Complement component 1q A chain
CaOX	Calcium oxalate
CCL2	C-C motif chemokine ligand 2 (MCP-1)
CCL3	C-C motif chemokine ligand 3
CCL5	C-C motif chemokine ligand 5
CCL6	C-C motif chemokine ligand 6
CCL9	C-C motif chemokine ligand 9
CCR2	C-C motif chemokine receptor 2
CCR5	C-C motif chemokine receptor 5
CCR6	C-C motif chemokine receptor 6
CD206 (MRC1)	Mannose receptor C-type 1
CKD	Chronic kidney disease
circRNA	Circular RNA
circACTR2	Circular RNA derived from ACTR2
CN	Crystal nephropathy
CSF1R	Colony-stimulating factor 1 receptor
CTGF	Connective tissue growth factor
CX3CL1	Fractalkine (C-X3-C motif chemokine ligand 1)
CX3CR1	C-X3-C motif chemokine receptor 1
CXCL1	C-X-C motif chemokine ligand 1
CXCL16	C-X-C motif chemokine ligand 16
CXCR2	C-X-C motif chemokine receptor 2
CXCR6	C-X-C motif chemokine receptor 6
DKD	Diabetic kidney disease
ECM	Extracellular matrix
ER	Endoplasmic reticulum
ESRD	End-stage renal disease
F13A1	Coagulation factor XIII A chain
FAO	Fatty acid oxidation
FIZZ1 (RELMα)	Found in inflammatory zone 1
Fn1	Fibronectin 1
FSGS	Focal segmental glomerulosclerosis
G6PD	Glucose-6-phosphate dehydrogenase
GSK3β	Glycogen synthase kinase 3 beta
HCK	Hematopoietic cell kinase
HIF-1α	Hypoxia-inducible factor-1 alpha
HK2	Hexokinase 2
IAM	Injury-associated myeloid (program)
ILC2	Group 2 innate lymphoid cell
IMQ	Imiquimod
IRF4	Interferon regulatory factor 4
IRI	Ischemia–reperfusion injury
JAK	Janus kinase
JAML	Junctional adhesion molecule-like protein
JMJD3	Jumonji domain-containing protein 3
KIM-1	Kidney injury molecule 1
LDHA	Lactate dehydrogenase A
Lgals3	Galectin-3
LN	Lupus nephritis
lncRNA	Long non-coding RNA
LOX	Lysyl oxidase
Ly6C	Lymphocyte antigen 6 complex locus C
M1	Classically activated (pro-inflammatory) macrophage
M2	Alternatively activated (pro-fibrotic) macrophage
M-CSF	Macrophage colony-stimulating factor
MCP-1	Monocyte chemoattractant protein 1 (CCL2)
MHC	Major histocompatibility complex
miRNA	MicroRNA
Mincle	Macrophage-inducible C-type lectin
MMPs	Matrix metalloproteinases
mTORC2	Mechanistic target of rapamycin complex 2
mtDNA	Mitochondrial DNA
NADPH	Nicotinamide adenine dinucleotide phosphate (reduced)
NEAT1	Nuclear paraspeckle assembly transcript 1
NF-κB	Nuclear factor kappa-light-chain-enhancer of activated B cells
NLRP3	NOD-, LRR-, pyrin domain-containing protein 3
NO	Nitric oxide
OXPHOS	Oxidative phosphorylation
PAMPs	Pathogen-associated molecular patterns
PDGF	Platelet-derived growth factor
PDGFβ	Platelet-derived growth factor-β
PDGFRβ	Platelet-derived growth factor receptor β
PFKFB3	6-phosphofructo-2-kinase/fructose-2,6-bisphosphatase 3
PGC-1β	PPARγ coactivator 1β
PINK1	PTEN-induced putative kinase 1
PI3K	Phosphoinositide 3-kinase
PKM2	Pyruvate kinase M2
PPARγ	Peroxisome proliferator-activated receptor gamma
PPP	Pentose phosphate pathway
PTEN	Phosphatase and tensin homolog
R-UUO	Reversible unilateral ureteral obstruction
RLDC	Repeated low-dose cisplatin
ROS	Reactive oxygen species
Sema3	Semaphorin-3 family
SIRT6	Sirtuin 6
SPP1	Secreted phosphoprotein 1 (osteopontin)
STAT1	Signal transducer and activator of transcription 1
STAT3	Signal transducer and activator of transcription 3
STAT6	Signal transducer and activator of transcription 6
ST2	Suppression of tumorigenicity 2 (IL-33 receptor)
STING	Stimulator of interferon genes
STZ	Streptozotocin
SUCNR1	Succinate receptor 1
TCA	Tricarboxylic acid cycle
TAK1	Transforming growth factor-β–activated kinase 1
TAZ	Transcriptional co-activator with PDZ-binding motif
TEC	Tubular epithelial cell
TG2	Transglutaminase 2
TGF-β	Transforming growth factor-beta
Thbs1	Thrombospondin-1
TLR4	Toll-like receptor 4
TNFR2	Tumor necrosis factor receptor 2
TRAF6	TNF receptor-associated factor 6
TRPV4	Transient receptor potential vanilloid 4
TREM2	Triggering receptor expressed on myeloid cells 2
UUO	Unilateral ureteral obstruction
VP	Verteporfin
Wls	Wntless (Wnt secretion mediator)
Wnt	Wingless/INT-1 signaling pathway
Wnt1	Wnt family member 1
Wnt3a	Wnt family member 3A
Wnt5a	Wnt family member 5A
Wnt7a	Wnt family member 7A
YAP	Yes-associated protein
YAP/TAZ	Yes-associated protein/transcriptional co-activator with PDZ-binding motif
